# Decellularized blood vessel development: Current state-of-the-art and future directions

**DOI:** 10.3389/fbioe.2022.951644

**Published:** 2022-08-08

**Authors:** Xinyu Wang, Vincent Chan, Peter R. Corridon

**Affiliations:** ^1^ Biomedical Engineering and Healthcare Engineering Innovation Center, Khalifa University, Abu Dhabi, United Arab Emirates; ^2^ Department of Immunology and Physiology, College of Medicine and Health Sciences, Khalifa University, Abu Dhabi, United Arab Emirates; ^3^ Center for Biotechnology, Khalifa University, Abu Dhabi, United Arab Emirates

**Keywords:** decellularization, recellularization, bioartificial, blood vessel, vascular tissue engineering, vascular replacement therapy

## Abstract

Vascular diseases contribute to intensive and irreversible damage, and current treatments include medications, rehabilitation, and surgical interventions. Often, these diseases require some form of vascular replacement therapy (VRT) to help patients overcome life-threatening conditions and traumatic injuries annually. Current VRTs rely on harvesting blood vessels from various regions of the body like the arms, legs, chest, and abdomen. However, these procedures also produce further complications like donor site morbidity. Such common comorbidities may lead to substantial pain, infections, decreased function, and additional reconstructive or cosmetic surgeries. Vascular tissue engineering technology promises to reduce or eliminate these issues, and the existing state-of-the-art approach is based on synthetic or natural polymer tubes aiming to mimic various types of blood vessel. Burgeoning decellularization techniques are considered as the most viable tissue engineering strategy to fill these gaps. This review discusses various approaches and the mechanisms behind decellularization techniques and outlines a simplified model for a replacement vascular unit. The current state-of-the-art method used to create decellularized vessel segments is identified. Also, perspectives on future directions to engineer small- (inner diameter >1 mm and <6 mm) to large-caliber (inner diameter >6 mm) vessel substitutes are presented.

## 1 Introduction

Vascular malfunctions often result in various maladies and are a leading cause of global mortality. Existing treatments include medications, rehabilitation, and surgical interventions. Medications have been developed to regulate cholesterol levels, ameliorate vasoconstriction, and control blood pressure. Alternatively, rehabilitation offers non-invasive approaches that incorporate active counseling, diet adjustments, and tailored exercise to support significant lifestyle changes. In comparison, surgical options, which are the most invasive, include angioplasty and stenting, atherectomy, endarterectomy, thrombectomy, and vascular bypasses. Vascular bypass is often used to treat severe conditions in large- (inner vascular diameter >6 mm) to small-caliber arteries (inner vascular diameter >1 mm and <6 mm). This autologous form of vascular replacement therapy relies on harvesting vessels from a patient’s arms, legs, chest, and abdomen to replace damaged vascular compartments (Crane and Cheshire, 2003).

Unfortunately, harvesting vessels from recipients can cause significant donor site morbidity, require expensive surgical procedures to repair lesions at the donor site, and often result in multiple post-surgery side effects like restenosis, aneurysms, infections, chronic paresthesia, dehiscence, and long-term pain (Thomas et al., 2013). The aesthetic outcomes of these surgical procedures may have severe social and psychological impacts on the patients, thus negatively impacting the patient’s quality of life and introducing significant financial burdens if further corrective and cosmetic procedures are required. Besides, around one-third of patients do not have proper and sufficient vessels for replacement owing to pre-existing vascular diseases, vein stripping, and previous surgical interventions. These issues have called for the need to develop transplantable vascular substitutes.

An ideal vessel replacement or equivalent should be biocompatible and biodegradable, while it possesses integrative and functional properties to support normal *in vivo* physiologic conditions such as pulsatile high-pressure blood flow, be capable of growth, and be cost-affordable. Scientists and surgeons have developed artificial vessel substitutes for decades to address this issue. Traditional prosthetic vascular analogs have been designed using Dacron®, polyethylene terephthalate, and Goretex®, expanded polytetrafluoroethylene (ePTFE) (Peck et al., 2012), and polyurethane (Kakisis et al., 2017). These synthetic materials were proved inferior to autologous vessels due to their rapid-decreasing patency, limited durability after 2 to 5 years, lack of compliance between grafts and native anastomosis, and increased risk of thrombosis and inflammation (Lin et al., 2018). In comparison, tissue-engineered vascular grafts (TEVGs) devised from adaptive manufacturing technologies like 3D printing, electrospinning, hydrogelation, cell seeding, as well as the application of various acellular/cellular substrates have been used to create artificial/bioartificial vascular conduits. Overall, it has been challenging to develop vessel substitutes that meet all the requirements simultaneously.

Nevertheless, vascular decellularization technologies have shown promise in meeting these challenges in recent years. This process removes cellular components from the native tissues to create an extracellular matrix (ECM)-rich, non-immunogenic vascular scaffold. Like their original forms, these natural vascular templates are believed to maintain an ability to support vasculogenesis during the reseeding process. Previous studies have outlined endothelial cells (ECs) compatibility with ECM scaffolds. Such compatibility is crucial for cellular adhesion, proliferation, and migration needed to create a functional and intact endothelium bordering the inner lumen of replacement vascular segments (Xu et al., 2014).

Various sources, including human and animal cadavers, have been identified as the supply of native vessels for decellularization. However, allografting and xenografting techniques can reintroduce issues related to immunogenicity. These issues will, in turn, give rise to the rejection of allografts and xenografts by primitive multicellular organisms mediated by phagocytic cells, coagulation proteins, and possibly by complement-like proteins (Cohen et al., 2021). Beyond these issues, it is interesting to note that some decellularized vessel products are currently evaluated for clinical use. However, clinical outcomes are unsatisfactory because of the graft-related thrombosis, infection, and aneurysm compared with other alternatives such synthetic conduits. Research is also being conducted to eliminate genetic remnants that give rise to rejection by using advanced gene-editing tools.

This review outlines the native vascular structure and then transitions to decellularization approaches and mechanisms, including physical, chemical, biological, and combinational treatments used to create decellularized vascular segments. The current state-of-the-art approaches used to create decellularized vessel segments are identified. Challenges and future perspectives to engineer small- to large-caliber vascular substitutes are also presented. Additionally, this review discusses various mechanisms behind this technology and outlines a simplified model for a replacement vascular unit based on innovative approaches to reinforce the structure and enhance physiological functions.

## 2 The native vasculature structure

Blood vessels are mainly categorized as arteries, veins, and capillaries, which nourish tissues and organs to sustain homeostasis. Generally, blood vessels can be characterized by their caliber, i.e., inner diameter (ID), as macrovessels (ID > 6 mm), small vessels (1 < ID < 6 mm), and microvessels (ID < 1 mm). Vessels within each of these three categories differ in cell composition, histological organization, and physiological function. However, all three types of blood vessels contain heterogeneous but well-orchestrated networks of ECM that regulate cellular and tissue functions and maintain vascular structural integrity. Adjacent thin endothelial cell layers found in the inner lumen are known for their endocrine and metabolic functionalities (Forslund and Metsärinne, 1992;Baumgartner-Parzer and Waldhäusl, 2001). ECs secrete substances that regulate vascular relaxation and contraction, angiogenesis, mitogenesis, blood clotting, platelet adhesion, and immune function.

The endothelium forms a crucial link between the cardiovascular and immune systems by expressing a range of innate pattern recognition receptors that allow ECs to respond to inflammatory stimulation, regulate immune cell recruitment, as well as extravasation into tissue targets. For example, ECs release endothelin, a potent vasoconstrictive polypeptide that exerts its major effects in both autocrine and paracrine regulations. This compound induces prolonged smooth muscle cell (SMC) contraction. Such secretions result in reduced blood flow, especially in renal and coronary circulations, and an increase in systemic blood circulation. Endothelin is also secreted in response to various biological stimuli, including vasoactive peptides such as angiotensin II, adrenaline, vasopressin, and thrombocytic products like transforming beta growth factor and thrombin.

SMC compartments within the vascular wall further control the vascular diameter, wall movement, and wall stiffness. From a classical perspective, these muscular regions can be divided into single- or multi-unit structures, as suggested by Bozler (Bozler, 1937;Bozler, 1970;Bozler, 1973). According to the Bozler model, single-unit (visceral) smooth muscle compartments are composed of cells electrically coupled by connexins through the formation of gap junctions. This unique conformation facilitates synchronous contraction, allowing the muscle layer to operate as an electrical syncytium. Whereas multi-unit smooth muscle layers, which are found in large blood vessels, lack, or rarely possess, gap junctions, differ from single-unit in that each smooth-muscle cell receives a synaptic input. This characteristic allows for the multi-unit smooth muscle to have much finer control. This division, which is based on motor nerve regulation and cellular electrical coupling, is predominately presented in the literature. However, this perspective does not fully describe the diversity of the tissue and is inconsistent with the experimental observations that have provided evidence of electrical coupling in all types of smooth muscles. A more recent division has been proposed to support phasic and tonic contractile responses that further outlines differences in cellular signaling and contractile kinetics.

The ECM is a microenvironment containing insoluble macromolecules that bind to its polymeric scaffold and soluble bioactive factors present in the liquid phase of this highly hydrated matrix, which regulate the behavior and functions of cells (Brown and Badylak, 2014). This microenvironment is an intricate multiphase network composed of an array of multidomain macromolecules organized in a cell/tissue-specific manner. Components of the ECM link or associate together to form a structurally stable composite, contributing to the mechanical properties of tissues and organs. At the same time, the ECM is a highly dynamic entity that is of vital importance, determining and controlling fundamental cellular functions, namely proliferation, adhesion, migration, polarity, differentiation, and apoptosis. Within these well-orchestrated networks are an abundance of glycoproteins, proteoglycans, glycosaminoglycans (GAGs), as well as structural proteins like collagen, elastin, and fibronectin, and laminin, which serves as the main component of the basal lamina (Davis and Senger, 2005; Hohenester and Yurchenco, 2013). These complexes define the two categories of molecules: glycosylated and fibrous proteins. Glycosylated proteins provide viscoelastic properties, support water retention, sustain osmotic pressures, and dictate proper fibrous protein organization (Iozzo and Schaefer, 2015). These qualities ensure tissue hydration that can withstand *in vivo* compressional forces (Yanagishita, 1993).

The fibrous extracellular networks of collagen and elastin provide the dominant mechanical responses of tissue. These two classes of fibrous proteins are secretory products from tissues and organs, and of these components, collagen is the most abundant fibrous protein and constitutes up to 30% of the total mass of mammalian proteins. Fibrous matrix proteins enable tissues to withstand high tensile and repetitive stresses without deformation or rupture. Collagen fibers have the greatest tensile strength and exhibit nonlinear responses when stretched, compared to elastin and fibronectin fibers, which are much more distensible (Williams et al., 2009). These structural ECM proteins work in tandem, whereby elastic fibers are tightly tethered to collagen fibrils to ensure that tissues resume their original shapes after compression or stretching. Moreover, during periods of extension, fibronectin fibers unfold to expose integrin-binding sites to facilitate cell adhesion. Such tension-activated cell-ECM adhesion is a central mechanism allowing cells to probe the mechanics of surrounding microenvironment (Merna et al., 2013). Once devoid of cells through decellularization, which isolates the extracellular matrix of the tissue from its native cells, it is hoped that the remaining matrices would offer a microenvironment naturally covered with molecular cues capable of initiating, for our purposes, vascular regeneration.

## 3 Decellularization approaches used to decellularize blood vessels

Decellularization offers a more efficient option to reduce the burdens of autologous grafting than other vascular tissue engineering (VTE) technologies, namely, those of a synthetic nature (Shakeel and Corridon, 2022). This approach generates far more accurate replicas of the ECM with superior bioactivity, immunogenicity, and biodegradability. This burgeoning technique relies on various physical, chemical, and biologic approaches or combinations of these individual approaches to rupture cellular membranes via immersive, agitative, and perfusive conditions.

Decellularization strategies vary widely based on distinct features of tissues, including structure, components, size, and thickness, as summarized in Table 1. Besides, antimicrobial treatments with penicillin, streptomycin, and amphotericin B are also applied to suppress antigen invasion (Jiménez-Gastélum et al., 2019). Theoretically, most cellular epitopes and antigens that trigger immune responses and contribute to implantation failure are ablated after vascular decellularization. As a result, decellularization leads to the optimized production of non-immunogenic vascular analogs that possess an ECM comparable to that of the native structure. Such an ECM scaffold can provide an environment conducive to cell integration/differentiation that is fit for *in vitro* vasculogenesis.

**TABLE 1:** A systematic summary of decellularization methods, their mechanisms of actions, and related references.

Decellularization technique		Mechanisms of action	References
Physical treatments	Free-thaw cycle	Thermal shock generated from repeated freezing and thaw cycles ruptures membranes	Rabbani et al. (2021)
	High hydrostatic pressure	Cold isostatic pressure disrupts cellular membranes within tissues	Funamoto et al. (2010)
	Electroporation	Microsecond- durational electrical pulses to enhance the cellular membrane permeability drastically	Sano et al. (2010)
	Supercritical fluid	Gases/liquids exist above critical pressure/temperature eliminate cell compartments within tissues	Di Maio et al. (2021)
	Subcritical fluid	Liquefied (subcritical) DME extracts lipids of tissues	Kanda et al. (2021)
	Immersion and agitation	Immerse tissues in chambers with decellularizing agents, and agitate tissues with a magnetic plate, ultrasound source, shaker, or an agitator attached to the end of the chamber.	Syed et al. (2014)
	Perfusion	The fluid (agent solution) passage through the circulation system to tissues/organs	He and Callanan, (2013), Corridon et al. (2017), Xu et al. (2017)
Chemical treatments	Acids and bases	Bases hydrolyze proteins to promote cellular debasement and acids lead to denaturation and protein function loss	Mendoza-Novelo et al. (2011), Angelova et al. (2018)
	Surfactants (ionic, non-ionic and zwitterionic)	Lyse cell membranes through protein crystallization, destabilizing, denaturing, targeting lipid-lipid interactions	Koley and Bard (2010)
	Chaotropes	Chaotropic agents disrupt the hydrogen bonding networks, van der Waals forces, and hydrophobic effects	Salvi et al. (2005)
	Osmotic stress	Hypertonic/hypotonic solution-induced osmotic stress/shock responses disrupt cell stability and interfere with the interaction between DNA and proteins	Fu et al. (2014)
Biologic treatments	Enzymatic approach	Enzyme-based processes that disrupt the bonds and interactions between nucleic acids and interacting cells through the disruptions of neighboring proteins and other cellular components.	Rieder et al. (2004), Fu et al. (2014)
	Combination of physical/chemical/enzymatic treatments	Physical, chemical, and enzymatic treatments are combined to optimize vascular decellularization	Row et al. (2017)

### 3.1 Physical treatments

Physical treatments involve thermal shock (freeze-thaw cycles), high hydrostatic pressures, supercritical fluids, pressure gradients, electroporation, ultrasound waves, vacuum technologies, immersion and agitation, and perfusion have been applied for decellularization (Rabbani et al., 2021).

#### 3.1.1 Free-thaw cycle

Thermal shock generated from freeze-thaw cycles, ranging from −87° to 37°, is applied to rupture cell membranes. Repeated freezing and thawing cycles help ice to form within the cytoplasm and cellular membranes. Various studies have established that damage to the plasma membrane occurs post-thaw stage, but there has been a debate on when the damage occurs during the cycle (Rabbani et al., 2021). Current mechanisms related to intracellular ice formation identify the plasma membrane as the primary injury site. During freezing, ice first forms in the extracellular water. This thermal change increases the ionic concentration in extracellular fluid and establishes an unregulated osmotic pressure gradient across the plasma membrane. This osmotic pressure gradient supports water efflux and cytoplasm dehydration and cell dehydration, while ice formation supports a phase transition leading to both mechanical and non-mechanical cellular damage.

This method can effectively remove cellular contents but may lead to minor ECM degradation and mechanical instability (Keane et al., 2015). Interestingly, the number of freeze-thaw cycles and the rates of temperature variation have not significantly affected decellularization efficiency, structure, or mechanical properties. Regarding blood vessels, Cheng et al. (2019). presented a study using freeze-thaw cycles to decellularize porcine carotid arteries, but this process was used in conjunction with chemical treatments of anionic and non-ionic surfactants. Microscopic and mechanical analyses unveiled well-maintained ECM morphology generated through the freeze-thaw cycles. Moreover, the resulting ECM was accompanied by the increase of arterial porosity after optimized use of both types of detergent. These results indicate that vascular scaffolds produced from the sequential use of freeze-thaw cycles and surfactants may serve as a promising option for VTE.

#### 3.1.2 High hydrostatic pressure

High hydrostatic pressure (HHP) treatment is based on the generation of cold isostatic pressure on tissues immersed in saline to generate acellular scaffolds. The technique disrupts the cellular membranes within the tissue, and the resulting debris can be removed from the ECM scaffold after rinsing with saline solution (Funamoto et al., 2010). From another mechanistic perspective, reports have shown that HHP can induce mitochondrial damage, demonstrate the overproduction of ROS, and ultimately apoptosis (Le et al., 2020). This option relies on the fact that cell membranes are disrupted by pressures of 100 MPa, while the three-dimensional architecture of the ECM is maintained even at 980 MPa.

A study using HHP of 980 MPa for 10 min successfully decellularized porcine arteries. Generally, HHP-decellularization efficiently removed cellular components and debris, preserved tissue’s mechanical properties, and suppressed structural damage. However, the amount of force applied must be precisely controlled in order to avoid nonideal structural alterations underlying the histological structure of the ECM. This process also supported xenogeneic transplantation, as HHP-decellularized arteries appeared to suppress immune responses and can be regenerated by recipient cell infiltration (Negishi et al., 2015). Allogenic transplantation showed that the acellular vascular scaffold endured arterial blood pressure and resisted luminal clot formation.

#### 3.1.3 Electroporation

Electroporation, a non-thermal and irreversible decellularization treatment, applies electrical pulses of microsecond duration to drastically enhance the cellular membrane permeability, presumably *via* the induction of nanoscale pores within lipid bilayers (Sano et al., 2010). Studies have shown that this technique is capable of decellularizing large volumes of tissue when combined with organ perfusion. Unfortunately, this application may be limited by the relatively small size of the electrodes used to induce the micropore formation and their propensity for generating tissue inflammation. However, this inflammatory response of immune system could be suppressed if decellularization was performed *in vivo* (Crapo et al., 2011). It should be noted that *in vivo* decellularization can lead to off-target and unwanted tissue degradation if not well-controlled.

Another form of poration utilizes mechanical wave-based ultrasound pulses to cleave intermolecular bonds, disrupt the cell membrane, and eliminate innate cellularities. Nevertheless, sonication-derived cavitation can severely damage tissue structure. Such effects were observed by Azhim et al. while studying the coupling between sonication and one of the most prominent chemical decellularization agents, SDS. Additionally, the porcine aorta was successfully decellularized by sonication at the luminal side and compared well to the results derived from treatment with SDS. The physical changes induced by this detergent resulted in the effective removal of DNA content and the retention of native mechanical properties (Sano et al., 2010). However, in some cases, immersion treatments need additional mechanical support in order to eradicate immunogenic components better. Hydrodynamic fluid forces induced by perfusion and alterations in temperature have remarkably reduced DNA residual content while maintaining the integrative ECM structures for the perseverance of mechanical functions (Zager et al., 2016).

#### 3.1.4 Supercritical and subcritical fluids

A supercritical fluid is a substance that can exist as a gas or liquid, and is used in a state above critical pressure and critical temperature when gases and liquids coexist (Di Maio et al., 2021). Supercritical fluids have also been used to eliminate cell compartments within tissues and to elute detergents from scaffolds after decellularization. Supercritical flow-based decellularization can impart minimal effect on tissue’s mechanical properties, but the elevated pressure to create such fluids can impair certain ECM components (Keane et al., 2015). Commonly applied parameters in this technique are based on critical temperature of 31.1°C and pressure of 7.40 MPa achieved in supercritical carbon dioxide (SC-CO_2_). This method is largely favored as no additional washing procedures are needed, and the intact structure and mechanical features are unchanged. For instance, porcine pericardial tissue, aortic and arterial tissues were decellularized with SC-CO_2_-based approaches leading to the complete removal of lipids, including a notable decrease of pro-inflammatory fatty acids, as well as efficient DNA removal (Gil-Ramírez et al., 2020). This technique generated ECM scaffolds with essential proteins (collagen Type I, collagen Type III, collagen Type IV, elastin, fibronectin, and laminin) and pivotal biological and growth factors (GAG, VEGF). Yet, the complex and expensive apparatus required to generate these zero viscosity fluids may eventually limit its application.

Likewise, another surfactant-free decellularization technique has been identified, in this case, using a subcritical fluid. This method uses liquefied (subcritical) dimethyl ether (DME). DME dissolved in water is not toxic to microorganisms and exhibits resistance to autoxidation, and can be used to extract lipids from wet porcine tissues by using subcritical DME at 23°C with a DME pressure of 0.56 MPa. Afterwards, DME will evaporate from the aorta at room temperature due to its low boiling point. Interestingly, results from this study by Kanda et al. (2021) clarified that although subcritical DME inefficiently removes cell nuclei, it can be used in tandem with DNase and saline/ethanol washing treatments to remove cell nuclei. Hematoxylin and eosin staining, and DNA analyses confirmed that most cell nuclei were removed from the aorta roughly 1–3 days after the initial DME treatment. DNA analyses uncovered residual DNA content of 40 ng dsDNA per mg ECM dry weight in the aortic scaffold. This value is lower than the standard value of 50 ng dsDNA per mg ECM dry weight proposed by Crapo et al. as the minimal criteria to assess the efficacy of decellularization (Crapo et al., 2011; Bruyneel and Carr, 2017). Overall, these results have demonstrated that subcritical DME in conjunction with DNase eliminates the need to utilize surfactants.

#### 3.1.5 Immersion and agitation

Immersive and agitative protocols are commonly used for hollow or less dense structures. This approach has been described for multiple tissue types, including blood vessels. The structure to be decellularized is immersed in chambers with decellularizing agents, and agitation is achieved with a magnetic plate, ultrasound source, shaker, or an agitator attached to the end of the chamber. It should be noted that the duration of the protocol of immersive/agitative approaches is a function of the tissue complexity, thickness, and density, as well as the detergent used, and the intensity and duration of applied treatment. Peristaltic pump-driven perfusion regimens can also be applied to enhance further the surface contact and homogeneity of the detergent-tissue interface (Syed et al., 2014).

#### 3.1.6 Perfusion

Perfusion can be conducted in antegrade and retrograde patterns for homogenously distributing the lysing agents throughout the organ via its underlying vasculature. With this confirmation, it is crucial to define tissue-specific flow rates that will ideally support fluid distribution and limit the development of elevated levels of pressure, which can be detrimental to thinner-walled and less resistant microvessels. Large and small animal model protocols have also been defined for blood vessel decellularization by using porcine abdominal aortic and carotid segments (Xu et al., 2017), and rat coronary arteries (He and Callanan, 2013). In contrast, perfusion-based decellularization protocols are widely recommended for whole organs with intact arterial and venous connections and, in some instances, hollow structures like vessels that can directly support the passage of fluid (Corridon et al., 2017).

### 3.2 Chemical treatments

Several chemical treatments for decellularization have been proposed, including acids and bases, surfactants (single ionic, zwitterionic, and non-ionic forms), chelators, exogenous enzymes, and hyper-/hypotonic solutions. These decellularization solutions have varied effects in standalone applications or class combinations, resulting in ECM products with different compositions. Such heterogeneity is observed in diverse levels of cellular component denaturation.

#### 3.2.1 Acids and bases

As an illustration, sodium hydroxide of sufficient strength can hydrolyze proteins in tissues to promote cellular debasement. Overall, alkaline treatments can introduce negative charges on the collagen molecules, which will create repulsive forces in tissues that drive substantial swelling suitable for pericardial decellularization (Mendoza-Novelo et al., 2011). Such treatment produced ECM scaffolds that possessed collagen structural networks, mechanical anisotropies, tensile moduli, tensile strengths, and strain maxima comparable to the native tissues. A study by Chuang et al. (2009) has also indicated that scaffolds generated by alkali-mediated decellularization efficiently removed native cells and DNA as revealed by histology and DNA analysis, respectively. This group aimed to develop tubular elastin scaffolds by using decellularization and removal of collagen from small-caliber porcine carotid arteries (diameters of approximately 5 mm). Alkaline extraction supported complete collagen removal while maintaining intact elastin and proteoglycans. Interestingly, the results from this study indicated that alkali-mediated decellularization is more conducive to cell repopulation than detergent-based methods.

Likewise, the combinations of reversible alkaline and nonionic surfactant (tridecyl alcohol ethoxylate) treatments retained general tissue structure and eliminated immunogenetic components. However, the inherent tissue swelling triggered a substantial loss of GAG content, and thus reduced viscoelasticity. Due to the acidic/basic properties of plasma membrane, pH directly affects membrane lipids, and substantial pH changes can induce lipid vesicle migration and extensive tissue deformation (Angelova et al., 2018). Acid oxidation can also lead to denaturation and loss of protein function. Specifically, acids can adversely alter protein stabilization by disrupting salt bridges and hydrogen bonds between the side chains, leading to denaturation. The process of protein oxidation frequently introduces new functional groups, such as hydroxyls and carbonyls, which contribute to altered biomolecular function and turnover.

For instance, peracetic acid has been used to create submillimeter-diameter vascular scaffolds. Yamanaka *et al.* decellularized rat tail arteries with an average inner diameter of 0.6 mm, using a combination of this acid and DNase I. Treatment with a 0.3% isotonic acid solution disrupted cell membranes and improved the decellularization efficiency in combination with the subsequent DNase treatment. A further illustration supporting combinative approaches outlined that the perfusion method was superior for cell removal compared to static immersion. However, histology showed that the perfusion of the DNase solution decreased ECM thickness, and this approach was not necessarily optimal for very thin vascular tissue decellularization.

*In vivo* application suggested that the reduction in mechanical strength of the decellularized scaffolds even after their luminal modification with peptides composed of collagen-binding and endothelial progenitor cell-binding sequences. These allogeneic implants remained patent for 2 weeks but unexpectedly ruptured after that. As often realized, this method identified the compromise between effective cell removal and maintenance of the ECM nature and highlighted the complexity in defining universal decellularization methods, the need to define protocols according to the type of target tissue, and its intended applications (Yamanaka et al., 2020).

#### 3.2.2 Surfactants

Surface-active agents such as surfactants, are widely used in biology for protein extraction from cell membranes for protein crystallization, as well as destabilizing, denaturing, and membrane permeabilizing agents (Koley and Bard, 2010). It can be argued that detergent-based decellularization is the most extensively applicable approach to induce cell lysis by disarranging the phospholipid membrane and uncontrolled membrane proration. Sodium dodecyl sulfate (SDS) and sodium deoxycholate (SD) are the most used anionic detergents. SDS is recommended for decellularization because of its efficient removal of cellular and genetic components and is well known for having strong electrostatic interactions with proteins, generated between aliphatic regions of the amino acids arginine and lysine and positively charged amino acids within primary protein structures supported by interactions of the hydrocarbon chains of the surfactant (Aguirre-Ramírez et al., 2021). Studies outlining the denaturing effect of SDS on globular proteins have identified that this process occurs at different stages depending on the concentration of the surfactant. Initially, SDS monomers bind to the protein to form groups up to a critical concentration for initiating denaturation. Further monomer binding alters the protein secondary structure. However, SDS is toxic and, damages tissues and organs' structure and mechanical properties, and efforts are needed to ensure its removal from scaffolds at the end of decellularization.

Concomitantly, SD can produce highly biocompatible scaffolds through the solubilization of cell membranes, resulting in decellularized matrices with better bioactivity, which is more suitable for cell seeding and growth. Although SD is less toxic, it can cause DNA agglutination on the surface of the scaffolds (Boccafoschi et al., 2017), reducing decellularization efficacy. On the other hand, cationic surfactants are more toxic than anionic surfactants and less effective as decellularization agents. Compared to anionic surfactants, cationic surfactants are positively charged and have a milder protein destabilization effect as they interact with negatively charged amino acid side chains, like aspartate (Peloso et al., 2015) and glutamate. Reports have shown that such interactions between cetyltrimethylammonium bromide, a popular cationic detergent, and lysozymes which are very hydrophobic and weakly electrostatic, do not cause a change in secondary protein structure but adversely alters the tertiary structure (Khan et al., 2015).

In contrast, nonionic surfactants targeting lipid-lipid interactions have minimum toxicity and are superior in preserving ECM architecture but inferior for removing DNA compared to anionic surfactants. Triton X-100 is one of the most widely used nonionic surfactants for lysing cells to extract proteins and other cellular organelles or permeabilize the living cell membrane for transfection. The nonionic surfactant Triton X-100 is frequently utilized to remove the remnant SDS in the perfusion process of organs (Corridon, 2021; Pantic et al., 2022). This surfactant has a disruptive effect on the polar head group as well as hydrogen bonds present within the lipid bilayer, altering the compactness and integrity of the lipid membrane. The insertion of a detergent monomer into the lipid membrane begins at low concentrations, which eventually disrupts cellular structures and eventually over permeabilizing the membrane as concentrations increase to support bilayer to micelle transitions. Aortic valvular scaffolds created using SD and Triton X-100 after formaldehyde fixation possessed mechanical properties close to those of the native valves (Singh et al., 2022). Combinations of Triton X-100 and SDS have also produced decellularized porcine carotid arteries with minimal destruction (Cai et al., 2021).

Zwitterionic constructs are ubiquitous in biological systems, from proteins to di-chain phospholipids within cellular membranes, and contain both positively and negatively charged chemical groups within their headgroups. Naturally occurring di-chain phospholipids are the main components of cell membranes. Such moieties generally show high solubility in water, broad isoelectric ranges, and high resistance to changes in the pH and ionic strength of the solution media, and their surfactants are known to form micelles in solution. Zwitterionic detergents exhibit the properties of both nonionic and ionic detergents and their tendency to denature proteins is more significant than that of nonionic detergents (Gratzer, 2019). However, the net-zero electrical charge on the hydrophilic groups of zwitterionic detergents protects the native state of proteins during the decellularization process (Keane et al., 2015). The molecule denoted by 3-[(3 cholamidopropyl)dimethylammonio]-1-propanesulfonate (CHAPS) is a notable zwitterionic surfactant that effectively eliminates protein-protein interactions and can match the cellular removal capacity of SDS (Keane et al., 2015).

Acellular scaffolds created by using CHAPS have better preserved ECMs, and lower remnant cell concentrations than non-ionic detergents (Gilpin and Yang, 2017). Explicitly, CHAPS-derived scaffolds retain more collagen, GAGs, and elastin than SDS ionic detergent while removing more than 95% of nuclear materials. It also revealed that CHAPS could be used in both perfusion and immersion processes with excellent maintenance of ECM proteins and structure but incomplete removal of cellular debris like cytoplasmic proteins (Sullivan et al., 2012). Again, the results obtained using this powerful denaturing agent are concentration-dependent, as higher CHAPS concentrations can disrupt collagen networks and basement membrane integrity, similar to higher SDS concertation solutions. For the vasculature, CHAPS-treated arterial tissues have unaltered collagen and elastin morphologies, and the collagen content remains comparable to that of the native artery. Protocols using another zwitterionic surfactant, tributyl phosphate or tri-n-butyl phosphate (TNBP), revealed that it also has a decellularization capacity similar to that of SDS by adequately eliminating tissue cellularities and keeping the intact ECM (Meezan et al., 1975; Gilpin et al., 2014). Moreover, collagen matrix, GAGs, and biomechanical properties (including tensile strength and elasticity) were not significantly altered. Although residual cytoskeletal proteins (e.g., vimentin) were not wholly removed, TnBP is a viable option that warrants further study for other tissue types.

Simsa et al. (2018) performed a systematic comparison of SDS, SD, CHAPS, and TritonX-100 for decellularizing the porcine vena cava and found that all surfactants can efficiently decellularize blood vessels. They presented data that outlined the acellular scaffolds generated with ionic and zwitterionic surfactants retained appreciable and comparable mechanical strengths, level of collagen preservation, and ECM integrities, but TritonX-100-based treatment required additional and long-term DNase application for complete decellularization. This group also examined the *in vitro* potential of human umbilical vein endothelial cells to adhere and proliferate on the luminal decellularized surface. Interestingly, the non-ionic surfactant-derived scaffolds showed the most remarkable efficacy for recellularization. These results build up those obtained by Kuna et al. a year earlier in their report outlining the decellularization of the human saphenous vein by using Triton X-100, TnBP, and deoxyribonuclease (DNase) and subsequent recellularization by perfusion of the peripheral blood and the endothelial medium.

#### 3.2.3 Chaotropes

Chaotropic agents disrupt the hydrogen bonding networks, van der Waals forces, and hydrophobic effects, specifically, non-covalent interactions in biological macromolecules, namely proteins (Salvi et al., 2005). Macromolecules attain more structural freedom that drives unfolding and destabilization through these agents while compromising the integrity of supramolecular assemblies (Hanai et al., 2020). Urea, a well-known chaotropic agent, denatures proteins and disrupts lipids and protein interactions to induce cell lysis, and chaotropic solubilization of proteins have prompted their evaluation of its potential as a decellularization agent (Wong et al., 2016). Over the years, urea has been used to produce ECM scaffolds from bovine umbilical veins (Wong et al., 2016).

#### 3.2.4 Osmotic stress

Hypertonic or hypotonic solutions induce osmotic stress or shock responses that disrupt cell stability and interfere with the interaction between DNA and proteins. The osmotic shock lyses cells, but it does not remove the cellular residues that it releases into the matrix. In particular, waste derived from DNA is of paramount importance in all decellularization processes due to the tendency of the nuclear material to remain tethered to ECM proteins (Fu et al., 2014). As a result, endonucleases, exonucleases, and other kinds of DNA degrading enzymes are used in conjunction with this approach to guarantee the elimination of nuclear components from ECM (Mendibil et al., 2020). Agents that rely on tonicity have limited utility as a standalone decellularizer method but can be applied to enhance the action of detergents.

As an additive mechanism, this technique generally involves the initial application of a hypotonic solution to stimulate uncontrolled cellular swelling that results in osmotic lysis. The unregulated inward movement of water may also enhance the uptake of detergents into the cellular membrane. After that, a hypertonic solution is applied to expel water from the cell and induce cellular dehydration and shrinkage that helps cellular death and detachment from the ECM. Finally, an additional nuclease enzymatic digestion is used to remove residual DNA. Osmotic shock-based decellularization strategies have created acellular templates of blood vessels already.

### 3.3 Biologic treatments

#### 3.3.1 Enzymatic approaches

Biologic treatments for decellularization are enzyme-based processes that disrupt the bonds and interactions between nucleic acids and interacting cells through the disruptions of neighboring proteins and other cellular components. Trypsin, nucleases (DNase and RNase), benzoase, and fetal bovine serum are widely used for decellularizing agents with low DNA, β-actin, and major histocompatibility complexes (MHC). These enzymatic agents are usually used with ethylenediaminetetraacetic acid (EDTA) or combined with surfactants to facilitate tissue or organ decellularization effectively (Rieder et al., 2004). These compounds have been widely investigated, and enzymatic treatments for decellularization can digest nucleic acids (DNA and RNA) and remove undesired ECM residues, as previously outlined for DNase activity (Rieder et al., 2004).

EDTA is a chelating agent that binds to calcium and prevents the integral proteins between cells from binding to one another. It is often used with trypsin, an enzyme that acts as a potent protease to cleave bonds between integral proteins of neighboring cells within a tissue. For instance, trypsin combined with EDTA cleaves cell-matrix adhesions to eliminate cellular and genetic materials after 24 h of treatment. This relatively short period appears to be appropriate for immunogenetic component abatement and mechanical property as well as vital surface molecule maintenance. However, trypsin/EDTA treatments can decompose salt- and acid-soluble collagens due to their higher affinity towards iron ions that stabilize this essential ECM protein (Zhang et al., 2016).

Furthermore, collagenases, proteases, and lipases have been used to remove cells in conjunction with other approaches. Collagenase-based treatments may be applied in cases that do not require a fully intact ECM. A study aimed to develop acellular-based vascular grafts from rodent iliac arteries outlined the application of collagenase IV detached the endothelium, and its combined use with detergents facilitated effective decellularization and eliminated MHC class I and class II antigens (Dall’Olmo et al., 2014). Lipase, e.g., phospholipase A2, treatments can hydrolyze the phospholipid component of tissues (Keane et al., 2015), while dispase, a well-known protease, would have prevented undesired cell aggregation and cleaved connections between fibronectin and collagen I and collagen IV to dislodge cells from the ECM (Fernández-Pérez and Ahearne, 2019; Nouri Barkestani et al., 2021; Neishabouri et al., 2022). Likewise, proteases like dispase and thermolysin (metalloproteinases) and lipase activities are not effective enough for complete delipidation and deproteination of the relatively large tissues and whole organs.

#### 3.3.2 Combined physical, chemical, and enzymatic treatments

Physical, chemical, and enzymatic treatments are combined to optimize vascular decellularization. This approach successfully developed functional, small vessels using a novel combination of zwitterionic, ionic detergents, and enzymatic treatments. Native samples were infused with CHAPS buffer followed by SDS solution. After that, to ablate unwanted cellular debris of these samples, they were incubated in a mixture of endothelial growth media-2 (EGM-2), 12% fetal bovine serum, and an enzymatic cocktail (Row et al., 2017). This innovative treatment has shown promising results for ECM retention, improved adhesion of HUVECs and SMCs *in vitro*, and reduced intimal hyperplasia (Gui et al., 2009). Apart from that, pressure gradients approach can adequately produce acellular templates from veins and other hollow structures in conjunction with an enzyme-mediated decellularizing process (Sierad et al., 2015).

Besides these treatments, decellularization processes have been developed to create large vessel scaffolds. The porcine abdominal aorta (approximately 20 mm in length) was decellularized with dichloroacetate DCA and DNase I, and the ECM was preserved to retain maximal tensile strength. Vessels harvested from the placenta were attached to a perfused capillary and assembled into a circulation system to lyse cells. Collagen and GAG retention facilitated EC attachment and mechanical property maintenance (Mancuso et al., 2014).

### 4 Current state-of-the-art for decellularized blood vessels

#### 4.1 Commercially available grafts

Commercially available decellularized vascular products include Artegraft® (bovine carotid artery), Solcograft (bovine carotid artery), ProCol® (bovine mesenteric vein), SynerGraft® (bovine ureter), MatrACELL® (decellularized ECM), decellularized human iliac or mammary veins, and Synergraft-processed cadaver vein allograft, which are clinically available (Kumar et al., 2011). For instance, Artegraft® is applied for hemodialysis, lower extremity bypass, and the treatment of traumatic arteries, whose advantages are reduced thrombosis, long-term durability, naturally biocompatibility, non-immunogenicity, and flexibility in multi-sizes for matching various host vessels (Pashneh-Tala et al., 2016).

ProCol® is a vascular bioprosthesis designed to form a bridge graft for vascular access when the previous prosthetic grafts have failed. This product supports vascular hysteresis to amplify pulsatile blood flow and possesses appreciable strength, durability, anastomotic compliance, and biocompatibility (Sharp et al., 2004). ProCol® has shown promising outcomes when used as a hemodialysis access line. Similarly, SynerGraft® (bovine ureter) is a foundation for the next generation of implantable biological tissues. For example, pulmonary and cardiac tissues treated with SynerGraft® were implanted into patients’ bodies without alloantibodies. Compared to standard cryopreserved allografts, SynerGraft® has excellent durability and better hemodynamics to suppress coagulation and maintain an integrative biological matrix.

This novel patented technology (SynerGraft®) created an underlying collagen matrix without epithelial cells, smooth muscle cells, and fibroblasts through denuding bovine ureters of cells by a series of hypotonic lysis and nuclease digestion (Das et al., 2011). These decellularized vascular products are processed by enzymatic/chemical methods, except for the SynerGraft® (bovine ureter) and SynerGraft®-processed cadaver vein allograft. Table 2 details the decellularization methods, indications, sizes, features, and other key properties of commercially available decellularized vascular products.

**TABLE 2:** A summary of commercially available decellularized vascular products, their associated applications, and clinical performance.

Vascular product	Product information	Approved indications	Clinical applications	Product advantages/Disadvantages
Artegraft®: bovine carotid artery(Harlander-Locke et al., 2014)	Decellularized steer’s carotid artery through physical and chemical treatments; ID: 4–8 mm; length:15–50 cm	Distal/segmental aorta replacement, arterial bypass/patch graft, arteriovenous shunt, and femoropopliteal bypass in lieu saphenous vein	Hemodialysis arteriovenous fistula grafting, salvage and repair, and lower extremity arterial trauma bypass	Reduced thrombus and patency rates compared to ePTFE, good saturability, and collagen matrix retains native cross-weave pattern with natural biocompatibility products available in multiple sizes to match host vessels
Cryovein®: human femoral vein (Fercana et al., 2014; Devillard and Marquette, 2021)	Small diameter grafts such as cryopreserved saphenous vein allografts	Saphenous veins, femoral veins, and femoral arteries for salvaging a localized prosthetic graft infection	Hemodialysis applications and extended lengths used to treat acutely ischemic limbs	Promising short-term, but extended thrombosis, poor 1-year patency, and aneurysmal degeneration led to rupture, calcification, and limited use
Cytograft, Lifeline^TM^ (Carrabba and Madeddu, 2018)	Vascular graft is a living conduit with the properties of a native vessel	Self-assembled cell-sheet of human fibroblast in a shape of vascular conduit	Arteriovenous shunt for hemodialysis	ECs were seeded in graft after devitalization, and constructed using patient cells, void of synthetic or exogenous material, but requires 6–9-month production time
Humacyte® (Devillard and Marquette, 2021)	Polyglycolic acid biodegradable scaffold with SMCs from deceased organ and tissue donors	Resulting bioengineered vessel is then decellularized to create conduit	Conducted its phase II clinical trials in patients with end-stage renal disease	63% permeability 6 months after implantation (in 60 patients), absence of immune response and lower infection rate than ePTFE grafts, yet permeability rate was 18% (< than ePTFE)12 months after implantation
MatrACELL®: decellularized ECM (Porzionato et al., 2018)	Decellularized human pulmonary artery patch	Anionic detergent, N-Lauroyl sarcosinate, and endonuclease	Pulmonary valve replacement	Retained biomechanical properties, biocompatible, and able to support cellular and vascular in-growth
ProCol®: bovine mesenteric vein (Schmidli et al., 2004)	Glutaraldehyde cross-linked bovine mesenteric vein; ID: 6 mm; length: 10–40 cm	A bridge graft for vascular access	Synthetic vascular grafts for patients who have at least one-time failed graft access	Improve pulsatile forward flow, durability, anastomotic compliance, minimal bleeding, and good degree of biocompatible
Solcograft®: bovine carotid artery (Lin et al., 2018)	Decellularized carotid artery cross-linked with adipyl dichloride	Vascular conduits for pediatric and adult use	Aortic, aortoiliac, carotid and vena cava replacement	Increased biochemical properties, no aneurysm, reduced infection and early thrombosis, and homogenous structure before and after implantation
SynerGraft®: cadaver saphenous/femoral veins (Pashneh-Tala et al., 2016)	Dimethyl sulfoxide-cryopreserved cadaver veins: Saphenous vein ID: 3–6 mm; length: 20–80 cm; Femoral vein ID: 6–15 mm; length: 10–30 cm	Saphenous vein bypass below knee in patients with infected fields that can’t generate fistulas	Arteriovenous access line, and bypass below knees for patients with infected fields and/or at risk of infection	Excellent durability and hemodynamics, and virtually eliminates presence of allogenic donor cells to maintain matrix structural integrity and need for anticoagulation

### 4.2 State-of-the-art *in vivo* applications

Successful examples of decellularized vascular conduits include multiscale components from diverse sources like carotid arteries from goats, and sheep, pulmonary aortas of sheep, rat abdominal/thoracic aortas, rat/porcine arteries, human umbilical arteries, porcine vena cava, saphenous/radial arteries, jugular veins from dogs, bovine ureters, arterial tissues of sheep, SIS, as well as human amnion membrane (Bertanha et al., 2014). Due to the limited clinical success of decellularized vessels on the market, there are not many commercial products. Nevertheless, these decellularized vascular replacements offer better results than alternative synthetic conduits (Lin et al., 2018). This fact is also another reason that restricts their use. On the contrary, their potential to replace synthetic devices supports the need for further research that can bring improved decellularized vascular segments to the market.

Endothelialization and SMCs repopulation can be observed in different layers of vessel structure within scaffolds. Acellular vascular grafts have been used in various models (e.g., sheep, goats, dogs, rats, mice, rabbits, and humans), providing patency rates of over 50% for at least 2 weeks. It is evident that the materials can vary according to their xenogeneic or non-xenogeneic nature, source locations within the cardiovascular system, and dimensions. Nevertheless, after generating decellularized ECM-derived scaffolds, some of those systems were reseeded with SMCs, ECs, and fibroblasts in different layers, while the others were implanted into anastomotic sites for endothelialization and remodeling. Histological results showed the most prolonged period for the convergence of the endothelialization is 8 weeks, and obvious recellularization happened at 6 weeks. During the remodeling process, no significant calcification, intima hyperplasia, graft dilation, rupture, or anastomotic aneurysm was detected (Bertanha et al., 2014).

In such cases, small-caliber (ID = 4 mm) decellularized ovine carotid arteries were grafted into 10 sheep and presented reendothelialization within 6 weeks with remarkably thicker neointima and apparent ECM reconstruction (Rijken et al., 2000). Besides, in 2013, decellularized rat thoracic aortas anastomosed to abdominal aortas maintained fully patent for 8 weeks, while only minute postoperative calcification was observed (Schneider et al., 2016). Similarly, Sakakibara and his colleagues implanted a decellularized rat artery (ID = 2–3 mm) to substitute the rat’s abdominal aorta, which functioned for 14 months with complete ECs coverage after 5 weeks, contractile function at 12 months, and formation of tunica intima and media by the end of their study (Xiong et al., 2013).

ECM-mimicking human amnion membrane (ID = 3.2 mm) interposed the rabbit carotid artery and reached complete patency rate at 4 weeks without notable diameter and blood flow deduction but markedly thickened midpoint wall (Conklin et al., 2002). Moreover, the decellularized - SIS (ID =10 mm) successfully replaced the vena cava in canines (Kleinert et al., 1996). Regarding the decellularizing of the vena cava, remarkably few studies have reported successful transplantation. An example from a recent thesis presented decellularized porcine vena cava created by using a combination of detergents (SDS, SDC, Triton X-100, and CHAPS)(Simsa, 2020) and DNase, along with immersive/agitative-based incubation. Six pigs were implanted with a decellularized vena cava created by this technique, and the grafts were void of thrombus formation and intima hyperplasia during the 5-week observation period. Furthermore, the researchers observed vascular cells on the third-day post-implantation.

Apart from the aforementioned vascular replacements, which have been decellularized directly from tubular vessel structures, several vascular constructs have been decellularized indirectly by perfusing cardiovascular organs. An example of such an approach relied on first decellularizing rat hearts by coronary perfusion with ionic detergents (Ott et al., 2008). In that study, aortas were cannulated for retrograde heart perfusion resulting in acellular, perfusable vascular components. These scaffolds were then repopulated with cardiac and endothelial cells that remained intact for up to 28 days. Additionally, other scientists have applied decellularization methods to intact pulmonary/systemic arteries to generate heart valves for transplantation. At the same time, they also obtained decellularized vascular analogs (Boccafoschi et al., 2017) with the hopes of defining clinical products.

With current models, clinical translation of vascular-derived grafts must originate from preclinical trials. Thus, it is essential to examine the feasibility of decellularized vascular constructs for *in vivo* applications during these periods. As for large-ID blood vessel replacements, commercial synthetic vascular conduits such as ePTFE and Dacron® are currently preferred and widely used compared to decellularized vascular grafts (Matsuzaki et al., 2019). Commercially available decellularized vascular grafts have limited performance due to the lack of cellularity upon implantation (Spark et al., 2008) and high production costs (Pashneh-Tala et al., 2016). Yet, synthetic vessel analogs on the market have been raising concerns because of unsatisfactory clinical outcomes resulting from graft-related thrombosis, infection, and aneurysm, which are even worse in small-ID vessel grafts (Sharp et al., 2004). Therefore, decellularized products may provide a viable alternative to creating small ID vascular replacements in the future.

According to a meta-analysis performed by Skovrind et al. on preclinical trials related to small-diameter tissue-engineered vascular grafts, the patency of these segments is significantly affected by recellularization, TEVG length/diameter, surface modification, and preconditioning (Skovrind et al., 2019). At the same time, the scaffold type was less impactful. Specifically, their analyses showed that TEVGs with a median ID of 3 mm, 4 mm, and 5 mm showed patencies of 63.5%, 89%, and 100%, respectively. Besides that, they also indicated that this measure was not enhanced through recellularization using SMCs, ECs, nor affected by the endothelial origin. The patency was most likely improved by the long-term (46–240 h) recellularization. Likewise, data analyses presented that the median TEVG length (5 cm) and median follow-up time (56 days) for *in vivo* decellularized analogs may lack the capacity for future direct clinical translation. In comparison, small-caliber (ID = 4 mm) decellularized ovine carotid arteries presented reendothelialization within 6 weeks (Rijken et al., 2000). These decellularized rat thoracic aortas anastomosed to abdominal aortas maintained complete patency for 8 weeks (Schneider et al., 2016) and appeared suitable as a clinical translation model. The potential of analogous decellularized rat arterial segments (ID = 2–3 mm) to substitute for the rat’s abdominal aorta and be functional for 14 months (Xiong et al., 2013) is further highlighted by such results. Overall, these evaluations have been established on preliminary preclinical data, and the precise requirements to support the transition to the clinic remain ambiguous. Nevertheless, this reminds us that future studies on TEVGs should incorporate endothelial recellularization and bioreactor preconditioning. We also recommend more detailed and precise guidelines for testing and reporting TEVGs in large animals. Meanwhile, to generate robust and reproducible outcomes and clinical translation, interstudy comparisons and multiple factors that affect the efficiency of vascular grafts should also be conducted (Skovrind et al., 2019).

### 4.3 Advances with *in vitro* models

Several achievements have been made using *in vitro* models. For example, such models have shown that decellularized biological scaffolds possess a propensity to be reengineered with ECs, SMCs, HUVECs, human iliac artery endothelial cells, fibroblasts, myofibroblasts, adhesion, and growth factors (Xiong et al., 2013). The scaffolds vary widely and include umbilical arteries, saphenous arteries, aortas, placenta vessel matrices, umbilical veins, and small intestinal submucosa (Xiong et al., 2013). After reseeding with various types of cells as mentioned above, these decellularized 3D matrices can provide an appropriate biological plateau to investigate vascular remodeling *in vitro*. Some examples of results obtained using these conduits are presented below.

Dahl *et al.* created flexible decellularized SMCs-based conduits with ID ≥ 6 mm or between 3 and 4 mm seeded using ECs, while Gui et al. re-endothelialized decellularized umbilical arteries with HUVECs(Gui et al., 2009). In addition, porcine pulmonary ECs can be grown on the decellularized porcine saphenous artery (ID ≈ 2 mm). These studies also showed that decellularized vascular scaffolds could regain appreciable function and reduce thrombogenicity by supporting cell regrowth, adhesion, migration, and differentiation (Fitriatul et al., 2017). Similar work also revealed that small-caliber acellular vascular grafts (ID < 4 mm) produced from portions of the placenta network could support HUVEC-based reendothelialization and phenotypic maintenance (Schneider et al., 2016). Uzarski et al. also discovered a way to drive the recellularization of human umbilical veins by using diverse groups of ECs (Dahan et al., 2017). SIS-derived scaffolds can also retain various angiogenic growth factors (such as basic fibroblast growth factor (bFGF) and vascular endothelial growth factor (VEGF) that proved to facilitate some degree of human EC retention, yet approaches to support a complete endothelium needs further exploration (Bader et al., 2000).

As for large–caliber veins, porcine and leporine vena cava (ID ≈13 mm) are commonly decellularized *in vitro*. For instance, researchers have used a combination of 1% Triton X-100, 1% TnBP, and DNase under three physical conditions: static immersion, agitation, and perfusion, and concluded that agitation or low-velocity perfusion with detergents are preferable for obtaining integrative and functional vena cava. The rabbit vena cava has also been decellularized with agitated SDS and SD incubation at a rate of 160 rpm at 37 °C, illustrating that both detergents produced effective results (Bader et al., 2000). These studies emphasize the potential for scaffold development that may ultimately drive neovascularization.

## 5 Future directions for decelluarizing vessels

Decellularization technologies have demonstrated an outstanding potential for developing bioartificial vascular substitutes based on ECM composition, ability to support remodeling, and propensity to minimize immunogenicity. Nonetheless, this technology still faces challenges and needs profound improvements to optimize structure, function, safety, and functionality. From a preclinical view, present decellularization methods may adversely alter the mechanical integrity of vessels and retain cellular and antigenic components that can induce immunogenicity. The inherent difficulty in accurately mimicking vascular structure is highlighted by the fact that we are still awaiting approaches that can generate complete tunica that can withstand *in vivo* conditions. In this case, strengthening the viscoelasticity and mechanical features within the decellularized wall with hydrogel-based approaches may be useful.

From a clinical perspective, most decellularized vascular grafts are used as larger-diameter replacements in hemodialysis or peripheral arterial bypasses with disappointing outcomes due to graft thrombosis, infection, and aneurysms (Bertanha et al., 2014). Apart from that, calcification and other forms of damage are present in patients after vessel replacement/repairment surgeries. Moreover, the autologous decellularized vascular grafting approaches are scarcely utilized, which drives the need for cadaveric allograft- and xenograft-derived templates, which still need to show their ability to present consistent clinical advantages over widely available and cost-effective synthetic counterparts (Khan et al., 2017).

Regarding the future directions of decellularized vasculature, researchers are inclined to combine other techniques such as hydrogelation, 3D printing/electrospinning, bioreactors, and decellularization/recellularization techniques to generate highly biomimetic scaffolds matching personalized regimens with high efficiency. It is currently difficult to generate an ideal decellularized scaffold by applying a single agent or technique from previous research. Therefore, it is believed that combinational technique-based decellularized techniques are the best way forward to facilitate VTE. For instance, hydrogels containing decellularized ECM (dECM) have been investigated and show a remarkable capacity for vascular cell reseeding compared to dECM scaffolds. Such cells can be primarily encapsulated in the hydrogel structure, facilitating recellularization. Also, these hydrogels can be used to create 3D printed or electrospun vasculature segments (Amirazad et al., 2022). 3D printing or electrospinning processes may coat the dECM with ECs and SMCs, and embed various factors like VEGF and platelet-derived growth factors (PDGF) to support vasculogenic multiscale substitutes development.

Reliable sources to obtain various cell types, ways to improve cell culture, and processes that drive differentiation and widescale recellularization are still needed. Efforts should be continued to enhance and potentially automate bioreactor systems with artificial intelligence to supervise regenerative processes, record morphological changes and functional performance of scaffolds, and continuously adjust the nutrient delivery and waste removal regimens (Wang et al., 2021). Finally, emerging organoid technologies can also be leveraged to provide insight into models that share complexity and scale with the microvasculature and provide a platform for examining vascular integration and interactions between vessels and tissues/organs via vascularization on a chip approach.

## 6 Simplified models for generating decellularized vascular units

**FIGURE 1:**
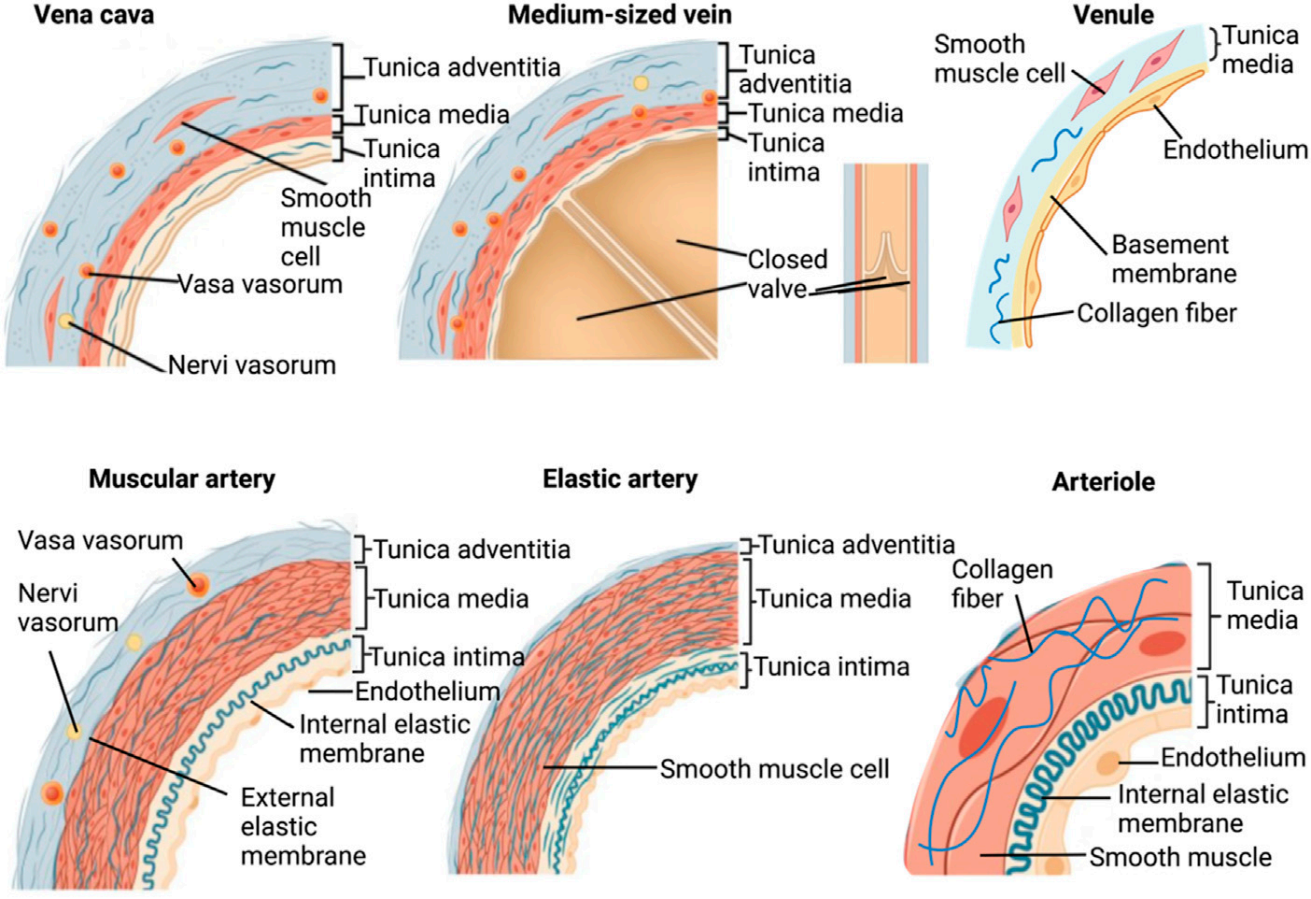
Structures of major native small- to large-caliber blood vessels.

Malone was the first investigator to demonstrate the potential of decellularized vessels for clinical application by showing that small-caliber arterial scaffolds grafted into canines could remain patent for roughly 3 months without an immune response (Sicari et al., 2014; Reis, 2019). Numerous studies have been conducted to define decellularization methods within the past 4 to 5 decades since these pioneering studies conducted by Malone presented the benefits of this technique in 1983. Since then, various other pioneering studies outlined above have supported decellularized vessel development, yet substantial gaps in knowledge still limit the extensive commercialization of decellularization-based VTE. As a result, further studies are required to support optimization techniques and ways to engineer small- to large-caliber vascular substitutes that truly mimic their native counterparts as shown in Figure 1. Therefore, we propose to define some essential components of acellular vascular graft models and approaches that may support the development of enhanced clinical substitutes in the near future.

The current state-of-the-art approach in the production of commercially available conduits highlights the limited structural composition of acellular compartments compared to native vessels. Native vessels possess diverse, complex, and well-defined layers, namely the tunica intima, tunica media, and tunica adventitia (or externa), with each having its own sub-layers/compartments which provide various structural and physiological functions. One can argue that instead of directly focusing on recrafting each independent layer, efforts within this field have been directed toward creating vascular walls that possess sufficient characteristics to support recellularization and *in vivo* pulsatile flow. Such research can simultaneously work to inhibit immune responses and thrombosis and, as technologies progress, help to realize the aim of extending this approach beyond the capabilities of synthetic grafts.

This simplified approach outlines vascular walls that rely on innate ECM-derived mechanical properties from the decellularized tunicas to support sufficient epithelial/endothelial cell regrowth, matrix stabilization, and functionalization. It is well known that the outmost layer of vessels is mainly composed of collagen-rich ECM. The functions of ECM provide signals that induce, define, and stabilize vascular phenotypes (Wagenseil and Mecham, 2009). Such characteristics must be maintained in the decellularized vessels since an intact, potent, and active ECM has a high potential for supporting long-term implantation. Furthermore, by using various decellularization methods, this model can be tuned to reinforce the structure and sustain adequate gas diffusion gradients and blood flow relative to the underlying contents and integrities of ECM fibers and glycoproteins. Perhaps such revised morphological and physiological properties are sufficient since the reduced cellular complexity no longer requires vasa vasorum to support additional nutrient and waste transport within the deeper layers of the vascular wall. There are two types of vasa vasorum: vasa vasorum externa and vasa vasorum interna. When the thickness extends beyond 1 mm, vasa vasorum are needed to support waste and nutrient exchange (Majesky et al., 2012). The former is found in the adventitia at its border with the media, and the latter originates from the luminal surface or the media and branches into the adjacent artery wall (Majesky et al., 2012).

Based on diffusion properties and considering what can be argued as the most complex vascular structure, the aorta, the existence of vasa vasorum makes diffusion more efficient by increasing surface area and reducing thickness, which Fick’s law of diffusion can be valid. This law states, $\dot{V}=\frac{DA\Delta P}{\Delta x}$ , which describes the time course of gas transfer $\dot{(V})$ , with respect to its partial pressure, (ΔP) and diffusion coefficient (D) , as well as the surface area (A) , and thickness (xΔ) of the membrane facilitating solute transfer. However, with decellularization, it is unknown how well the structural components remain within the vasa vasorum. This issue can be further complicated across different species, as mice and other mammals with ≤29 elastic lamellar do not have vasa vasorum (Wagenseil and Mecham, 2009; Billaud et al., 2018), which indicates that choosing decellularizing sample species plays a vital role. Thus, larger mammals with >29 medial lamellar are preferable for exploring the vasa vasorum, and it might be generated through 3D printing and electrospinning since these two technologies focus on the microvasculature and can be investigated with powerful vascular *in vivo* modalities like micro-computed tomography (Zagorchev et al., 2010) and intravital microscopy (Eriksson, 2011; Corridon et al., 2021). Furthermore, this issue may be counteracted by the increased degree of ECM porosity that can be achieved with decellularization to support nutrient exchange with reseeding models.

It is also essential to consider various versatile molecules within the native ECM, such as GAGs (Dewey et al., 2021) and VEGF (Liang et al., 2001), for promoting vasculogenesis. GAG chains are attached to proteoglycans which, based on the physicochemical characteristics of the glycosaminoglycan component, provide hydration and swelling capacities that allow tissues to withstand compressional forces (Yanagishita, 1993). The large proteoglycans interact with hyaluronic acid, forming an extensive, interconnected polymeric network in the extracellular space (Davison et al., 1995). The types and amount of GAG chains linked to proteoglycans can affect the matrix assembly (Berry and Greenwald, 1976) and porosity (Corridon et al., 2006). Meanwhile, growth factors like VEGF can support the regrowth and differentiation of endothelium, as observed in decellularized models (Lin et al., 2018) and VEGF-coated stents to accelerate re-endothelialization (Swanson et al., 2003). These two factors may ultimately enhance the structural and functional abilities of the aggregated decellularized tunica.

Autonomic innervation also plays a vital role under a complex homeostatic mechanism observed within native blood vessels that helps to regulate vascular diameter. For example, in addition to the intrinsic vessels (vasa vasorum), the adventitia houses collections of nerve fibers called nervi vasorum, i.e., nerves of the vessel, which modulate vascular vasodilatative and vasoconstrictive capacities through the incorporation of SMCs. These muscular cells within the tunica media of the native aorta (and in media and adventitia of vena cava) deposit collagen and elastin fibers that account for tensile strength and distensibility, respectively. Previous studies suggest that less than 10% of collagen bears physiological pressure, while once the pressure rises, the collagen fibers are recruited to support the passive wall tension and limit aortic distention, making vessels progressively distensible (Davis, 1993). However, as pressures exceed the standard physiological threshold, collagen fibers automatically realign to limit radial expansion, and help elastic fibers recoil. This tightly regulated mechanism is lost in denervated decellularized vessels. As a result, retaining ECMs with optimal collagen and elastin fiber networks for vascular structures now devoid of nervi vasorum, SMCs, and thus, their innate regulatory capacities (Corridon, 2021).

**FIGURE 2:**
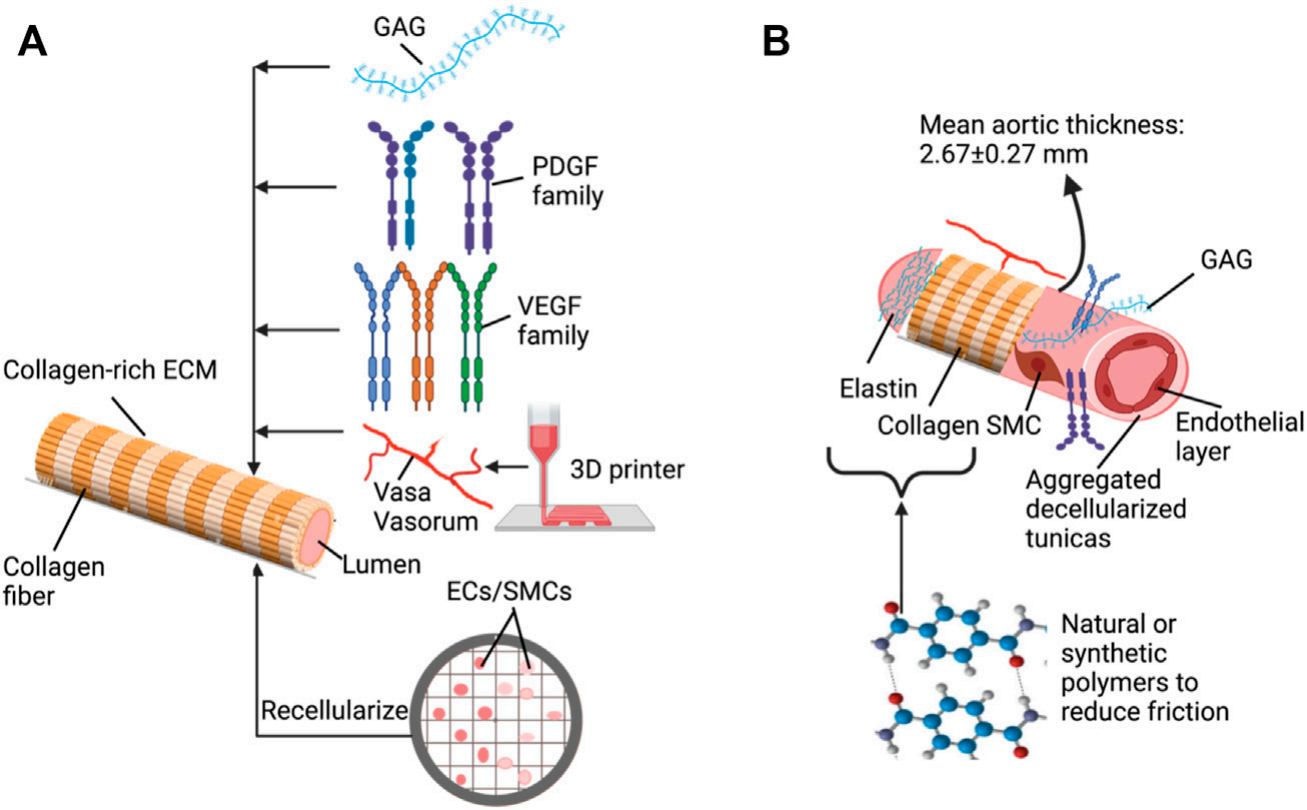
Key elements and approaches to support the development of decellularized vascular grafts: **(A)** basic decellularization vessel model that can be tuned with various additives **(B)** using combinative approaches to generate improved vascular substitutes.

Finally, ensuring that the anticoagulant nature of the decellularized vascular lumen mimics what is produced by the native endothelium will aid in preventing thrombosis. The endothelium achieves this by providing a surface that discourages the attachment of cells and clotting proteins (Yau et al., 2015). This layer also modulates vascular tone, blood cell aggregation, and deformability (Simmonds et al., 2014). Such traits impact the release of endothelial mediators in response to chemical (production of oxygen free radicals and nitric oxide that alter vascular tone) and physical (changes in blood viscosity that alter shear stress) stimuli (Forconi and Gori, 2013). These effects can be described by the Hagen-Poiseuille’s law, which states, $Q=\frac{\pi r^{4}\Delta P}{8l\eta }$ , where the rate of blood flow (Q) is influenced by blood pressure (ΔP) and viscosity (η) , as well as vessel length (l) and radius (r) . We have recently used this relationship to describe flow rates through the decellularized renal artery and vein under normothermic and hypothermic conditions in a whole decellularized organ model with controlled pressures and varying viscosities (Corridon, 2021). Thus, this relationship may provide a way to optimize the replacement graft diameter and length for a given grafting procedure. Another aspect that can stem from this approach is the inability to regulate the wall thickness of decellularized vessel products. The overall approach, as summarized in Figure 2, may identify the necessary and sufficient decellularized wall components capable of supporting gas diffusion and withstanding debilitating blood viscosities using global mean aortic widths, 2.67 ±0.27 mm (Liu et al., 2015), as a starting point. In particular, the model may also support combinative decellularization technologies to tune luminal surfaces with natural or synthetic polymers to reduce friction between moving blood and stationary acellular walls in large- to small-caliber replacement vessels.

## 7 Conclusion

This review discussed different methods for decellularizing multiscale blood vessels. Theoretically, all of these methods can create acellular templates. Different decellularization strategies generate vessel substitutes with viable mechanical features, structures, and levels of patency, and combinational treatments have so far provided the best results. Ideally, scientists have to minimize immunogenicity and maximize physiological performance. So far, we have gained tremendous insight into how decellularizing agents impact native structures, which has led to successful cadaveric transplantation and xenotransplantation models, some of which have been translated to the clinical applications. These achievements, in turn, have spawned interest in generating commercially available vascular substitutes derived from natural materials to replace existing synthetic devices. However, several challenges must still be overcome to support this progression, including identifying unified decellularization and recellularization protocols and improving the basic tenets needed to support long-term implantation. Perhaps solutions to these major challenges may come from other combinative approaches that utilize emerging strategies. Altogether, the future appears promising as we strive to develop the state-or-the-art in VTE using decellularization technologies.
